# Improved closure of the global mean sea level budget from observational advances since 1960

**DOI:** 10.1126/sciadv.aea0652

**Published:** 2026-05-20

**Authors:** Huayi Zheng, Lijing Cheng, Sönke Dangendorf, Benoit Meyssignac, Anne Barnoud, Kevin E. Trenberth, John T. Fasullo, John Abraham

**Affiliations:** ^1^ State Key Laboratory of Earth System Numerical Modeling and Application, Institute of Atmospheric Physics, Chinese Academy of Sciences, Beijing, China.; ^2^ University of Chinese Academy of Sciences, Beijing, China.; ^3^ Department of River-Coastal Science and Engineering, Tulane University, New Orleans, LA, USA.; ^4^ Université de Toulouse, LEGOS (CNES/CNRS/IRD/UT3), Toulouse, France.; ^5^ Magellium, Ramonville-Saint-Agne, France.; ^6^ NSF National Center for Atmospheric Research, Boulder, CO, USA.; ^7^ Department of Physics, University of Auckland, Auckland, New Zealand.; ^8^ Department of Atmospheric and Oceanic Sciences, University of Colorado Boulder, Boulder, CO, USA.; ^9^ School of Engineering, University of St. Thomas, St. Paul, MN, USA.

## Abstract

Balancing the global mean sea level (GMSL) budget is essential for understanding sea level changes. Large uncertainty after 1960 is reduced by accounting for recent observational advances. Budget closure occurs within 0.18 millimeters per year for all periods analyzed (1960–2023, 1993–2023, and 2005–2023). Trends for these three periods are 2.06, 3.41, and 3.94 millimeters per year, revealing an increase in the rate. The annual residual between observed GMSL and the sum of contributions is only between −13 and 10 millimeters since 1960 and ±5 millimeters after 2005. Further, the GMSL acceleration budget is now closed. The principal drivers for the GMSL trend (acceleration) since 1960 are 43% (41%) from thermosteric ocean expansion, 27% (9%) from glacier melting, 15% (16%) from Greenland, 12% (13%) from Antarctic, and 3% (21%) from land water storage. Results highlight the importance of data processing and bias correction techniques in tracking GMSL and its contributions.

## Teaser

Recent observational advances lead to a closure of trend and acceleration budgets across various time periods.

## INTRODUCTION

Accelerating sea level rise poses substantial hazards to low-lying coastal regions (*1*, *2*). Understanding the causes of sea level rise is indispensable for projections of future sea level changes (*3*, *4*) and supports climate adaptation and mitigation efforts. Global mean sea level (GMSL) rise is driven by two main processes: barystatic changes in ocean mass and (thermo-)steric expansion. Barystatic sea level is associated with freshwater influx from glaciers (*5*, *6*), the Greenland and Antarctic ice sheets (*7*), land water storage (*8*, *9*), and atmospheric water vapor. Thermosteric sea level change is a volumetric change driven by ocean expansion or contraction from temperature variations (*10*).

Agreement between the sum of all these known contributions and GMSL observations would demonstrate a good physical understanding of the causes for sea level rise, while discrepancies suggest that there are unresolved systematic errors in observations of either GMSL or individual contributions (*11*–*13*). The sea level budget provides an important constraint for future projections from climate model simulations and climate policies (*4*). Moreover, a closed sea level budget is crucial for reliably estimating the Earth’s energy imbalance (EEI). About 90% of the excess energy is stored in the ocean. Ocean warming and EEI can be derived indirectly from the differences between GMSL and barystatic sea level (*14*, *15*).

State-of-the-art observations as reported in the Sixth Assessment Report of the Intergovernmental Panel on Climate Change (AR6) (*16*) indicate that the trend of the sum of contributions (2.00 ± 0.49 mm year^−1^, with a 90% confidence interval) is lower than the observed GMSL rise (2.33 ± 0.79 mm year^−1^) over 1971–2018. Although within the uncertainty range, a difference of 0.33 mm year^−1^ is larger than the key individual contributions from the Greenland Ice Sheet (0.25 ± 0.09 mm year^−1^), land water storage (0.15 ± 0.20 mm year^−1^), and the Antarctic Ice Sheet (0.14 ± 0.23 mm year^−1^) over the same period.

Thanks to the development of the global observation system (such as satellite altimetry, satellite gravimetry, and Argo), the residual of the GMSL budget has been reduced to ∼0.08 mm year^−1^ between 2005 and 2018 (*16*). However, a considerable residual has occurred again since ∼2015, with 1.54 ± 0.51 mm year^−1^ for the 2016–2020 period, and the residual time series exceeds ±5 mm range (*17*, *18*), which raises doubts about the current observation system. Multiple systematic drifts or biases in instruments have been identified to contribute to the budget residual, including a positive Argo salinity drift (*12*), the potential long-term drift in microwave radiometers on Jason-3 (*19*), ocean temperature biases from several instruments (*10*, *20*–*23*), and “conservative biases” in the traditional ocean temperature gap-filling approach (*24*). The budget residual after 2015 has not yet been fully solved.

It is well established that the GMSL rise is accelerating (i.e., the rate is increasing) (*25*–*27*), owing to the acceleration of ocean warming (*28*, *29*), mass loss of mountain glaciers (*5*, *6*) and ice sheets (*30*), and decreasing land water storage (*31*). However, the extent to which these factors contribute to the acceleration of GMSL over different time periods has been unclear. Dangendorf *et al.* (*26*) constructed an acceleration budget since 1960 using tide gauge reconstructions and estimates of the barystatic component, with the steric contribution inferred from a reduced spatial optimal interpolation applied to the residual between the two. The budget suggests that the steric component is the major driver of acceleration; however, the budget is not fully independent of the individually measured components, given that the barystatic component serves as a prior. Nerem *et al.* (*32*) identify Antarctic Ice Sheet mass loss as the major acceleration contribution, accounting for 45%, by using glaciers, ice sheets, and land water storage estimates based on Gravity Recovery and Climate Experiment, and its follow-on (GRACE/GRACE-FO) from 2002 to 2017, compared with 1993 to 2017 GMSL and thermosteric sea level changes. Nevertheless, the acceleration of barystatic components since 2002 might not fully represent the change since 1993 (Table 1) because of the presence of strong interannual variability (*8*, *33*, *34*).

**Table 1.** Trends and accelerations for observed GMSL and its individual contributors. Trends (in mm year^−1^) and accelerations (in mm year^−1^ decade^−1^) with their corresponding 90% confidence intervals for observed GMSL and its individual contributors based on annual changes. Uncertainties of the sum of contributions (i.e., the sea level budget) represent the root mean squares of component errors, assuming that the associated errors of individual components are independent.

	1960–2023 Trend (mm year^−1^)	1960–2023 Acceleration (mm year^−1^ decade^−1^)	1993–2023 Trend (mm year^−1^)	1993–2023 Acceleration (mm year^−1^ decade^−1^)	2005–2023 Trend (mm year^−1^)	2005–2023 Acceleration (mm year^−1^ decade^−1^)
Thermosteric sea level	0.81 ± 0.12	0.35 ± 0.07	1.46 ± 0.21	0.10 ± 0.15	1.55 ± 0.28	1.15 ± 0.33
Glaciers	0.51 ± 0.03	0.08 ± 0.01	0.66 ± 0.04	0.13 ± 0.05	0.73 ± 0.03	0.14 ± 0.02
Greenland Ice Sheet	0.29 ± 0.04	0.14 ± 0.01	0.59 ± 0.04	0.20 ± 0.05	0.70 ± 0.05	−0.25 ± 0.02
Antarctic Ice Sheet	0.23 ± 0.03	0.11 ± 0.02	0.44 ± 0.05	0.09 ± 0.06	0.51 ± 0.07	−0.26 ± 0.03
Land water storage	0.05 ± 0.25	0.18 ± 0.07	0.32 ± 0.09	0.05 ± 0.18	0.35 ± 0.12	−0.05 ± 0.44
Atmospheric water vapor			−0.05 ± 0.00	0.00 ± 0.00	−0.05 ± 0.00	0.07 ± 0.00
Sum of contributions	1.89 ± 0.28	0.86 ± 0.10	3.42 ± 0.24	0.57 ± 0.25	3.79 ± 0.32	0.80 ± 0.55
GMSL	2.06 ± 0.35	0.72 ± 0.18	3.41 ± 0.31	0.83 ± 0.49	3.94 ± 0.31	0.84 ± 0.66

**Fig. 1.**
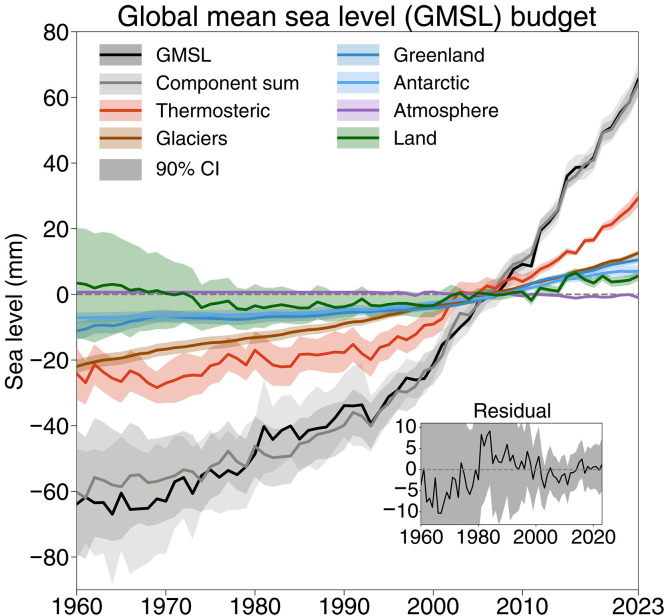
Time series of observed GMSL and individual contributions relative to 1991–2020. The inset in the panel shows the residual of the budget (observed GMSL minus the sum of components). Shading indicates the 90% confidence intervals (CI).

As such, the closure of the GMSL trend and acceleration budget remains a challenge. However, since AR6, updated estimates involving many processes have been published for GMSL and its components (table S1). The remaining question is the extent to which these independent developments contribute to the closure of the GMSL budget. Here, we use the most recent data developments and community estimates to reassess the budget of GMSL trends and accelerations. The GMSL trend and acceleration budgets (Fig. 1) are analyzed over three distinct periods: long term (1960–2023, accounting for the length of the reliable in situ thermosteric sea level data), altimetry era (1993–2023, the satellite altimetry data start from 1993), and the Argo era (2005–2023) (Table 1). The central estimates of trends and accelerations are calculated using the ordinary least squares (OLS) method (see the “Methods” section) based on annual changes, but other approaches used in previous studies, such as the locally weighted scatterplot smoothing (LOWESS) method (*28*) and Delta method (*35*), are also tested (table S2). Trend and acceleration uncertainties are calculated with a unified approach based on the error variance-covariance matrix accounting for data uncertainty and its characteristics (*25*).

## RESULTS

### Long term (1960–2023)

GMSL is estimated from a composite of tide gauge reconstructions (1960–1992) and satellite altimetry (1993–2023) (see the “Methods” section). For 1960–2023, the composite time series indicates a GMSL rise of 2.06 ± 0.35 mm year^−1^. The sum of contributions indicates a linear trend of 1.89 ± 0.28 mm year^−1^, to which thermosteric sea level accounts for the majority with 43%, followed by glacier mass loss with 27%, the Greenland Ice Sheet mass loss with 15%, the Antarctic Ice Sheet mass loss with 12%, and land water storage variations with 3%. The residual between observed GMSL and the sum of contributions is 0.17 mm year^−1^ (0.12 mm year^−1^ for 1971–2018), much smaller than in previous studies: for instance, 0.33 mm year^−1^ for 1971–2018 by AR6 and 0.26 mm year^−1^ for 1958–2018 by Frederikse *et al.* (*36*) (hereafter F2020). Because global observations of atmospheric water vapor before 1993 are compromised by heterogeneities (*37*, *38*), this component is excluded from the budget for this period.

For acceleration (calculated by a quadratic linear fit), the GMSL composite suggests an acceleration of 0.71 ± 0.18 mm year^−1^ decade^−1^ for 1960–2023, with individual contributions summing to 0.86 ± 0.10 mm year^−1^ decade^−1^. This agreement gives confidence that the current estimates can robustly quantify the causes of GMSL acceleration over the past 60 years. Thermosteric sea level is the major contributor, explaining 41% of the acceleration. Land water storage variations, Greenland Ice Sheet mass loss, Antarctic Ice Sheet mass loss, and glacier mass loss contribute 21, 16, 13, and 9%, respectively.

To investigate the reason for the better GMSL closure, we compare the estimates with AR6 for 1971–2018 (table S3). Our GMSL composite record is 0.14 mm year^−1^ lower than that in AR6 (Fig. 2A). The tide gauge reconstructions used in this study are based on the three most recent estimates (*36*, *39*, *40*), which now all use prior information about spatial variability in relative sea level due to vertical land motion and barystatic fingerprints of gravitation, rotation, and deformation. AR6 adopted the tide gauge reconstruction estimate from Palmer *et al.* (*35*), which included five reconstructions (*26*, *36*, *41*–*43*). One of these members (*42*) corrects only for vertical land motion due to glacial isostatic adjustment (GIA), leading to an overestimate of GMSL (*39*, *43*). We also note that our three considered GMSL reconstructions represent updates to those used in Palmer *et al.* (*35*).

**Figure:**
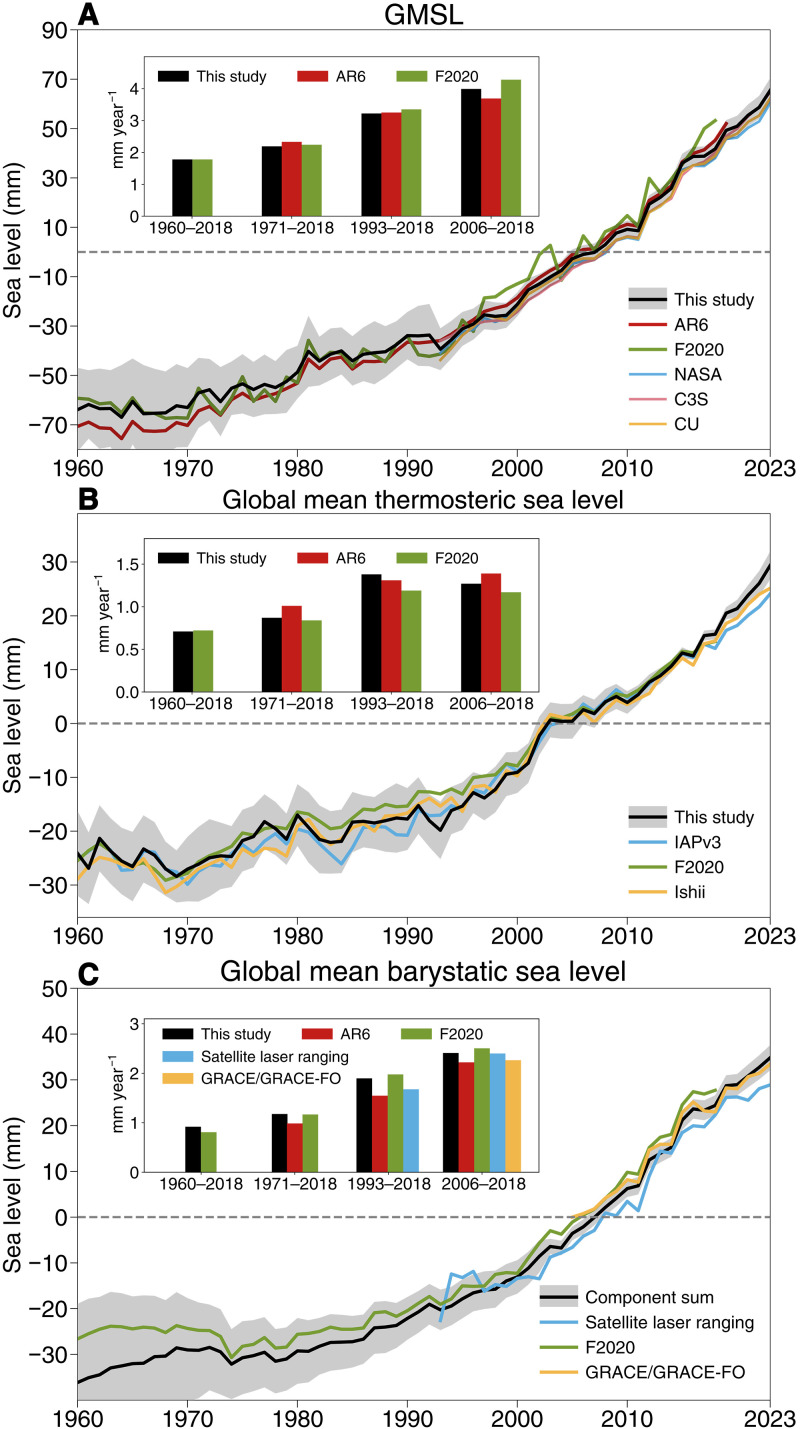
Fig. 2.Comparison of the GMSL components derived in this study with other datasets. Comparison of GMSL (**A**), global mean thermosteric sea level (**B**), and global mean barystatic sea level (**C**) from this study with estimates from other datasets. The black time series indicates data from this study, and shading represents a 90% confidence interval. The inset panel shows the trends from AR6, F2020, and this study over the overlapping periods (1960–2018, 1971–2018, 1993–2018, and 2006–2018). The time series are relative to the 1991–2020 mean.

Budget estimates based on individual GMSL reconstructions (without combining them with satellite altimetry after 1993) can be found in fig. S1. When using the reconstruction from Dangendorf *et al.* (*40*), the budget shows the smallest residual. This is because the approaches used by Wang *et al.* (*39*) (reduced spatial optimal interpolation with EOF0, hereafter RSOI) and F2020 (the virtual station technique) are basically weighted averages of the tide gauge records (*44*) and are therefore affected by sampling uncertainty. The latter is improved by Dangendorf *et al.* (*40*) by coupling the Kalman Smoother with the RSOI (without EOF0). As a result, around 1981/82, Wang *et al.* (*39*) and F2020 have a large interannual peak that initiates a consistent bias with the budget components in the preceding years.

### Altimetry era (1993–2023)

During the 1993–2023 period, GMSL with a correction for wet troposphere correction (WTC) based on climate records (*19*) and ocean bottom deformation (*13*) has a linear trend of 3.41 ± 0.31 mm year^−1^ from satellite altimetry, in excellent agreement with the sum of contributions (3.42 ± 0.24 mm year^−1^). The contributions of each component to the GMSL trend are as follows: Thermosteric changes contribute 43%, glacier mass loss 19%, Greenland Ice Sheet mass loss 17%, Antarctic Ice Sheet mass loss 13%, land water storage changes 9%, and atmospheric water vapor changes −1%.

Satellite altimetry indicates a GMSL acceleration of 0.83 ± 0.49 mm year^−1^ decade^−1^. The value is lower than previous altimetry studies (1.2 ± 0.5 mm year^−1^ decade^−1^ over 1993–2022) based on the TOPEX/Poseidon and Jason altimetry missions (*25*, *45*) because of the WTC adjustment. The WTC based on Jason-3 microwave radiometers is likely subject to a spurious drift. Thus, we adopt the WTC derived from climate data records (*19*). The new WTC decreases the acceleration by 0.15 mm year^−1^ decade^−1^. The acceleration after the adjustment of WTC is closer to the results of 0.93 ± 0.14 mm year^−1^ decade^−1^ over 1991–2019 based on ERS-1 and ERS-2 and Envisat altimetry missions (*46*). The uncertainty of acceleration (0.49 mm year^−1^ decade^−1^) is a little smaller than that in previous studies (*25*, *27*) (about 0.60 mm year^−1^ decade^−1^) because the intermission offset uncertainties of satellite altimetry are reduced, benefiting from the tandem flight phase between two successive satellites (*47*, *48*).

The acceleration of the barystatic component sum is 0.47 ± 0.20 mm year^−1^ decade^−1^. Thermosteric sea level contributes 0.10 ± 0.15 mm year^−1^ decade^−1^. The weaker acceleration compared with that of 1960–2023 is likely associated with the volcanic Mt. Pinatubo eruption in 1991, which led to an ocean cooling lasting several years, followed by a bounce back in the late 1990s (*49*). Thus, the ocean warming rate is larger in the 1990s than in the early 2000s, resulting in a deceleration over that period. Then, with strong greenhouse gas forcing, recent aerosol emissions reductions (*50*), and energy redistribution driven by atmospheric and ocean circulation changes (*51*), the ocean warming rate increases again after ∼2010. These forcing changes result in large uncertainty in the acceleration calculation, which is sensitive to the start and end point choices.

To highlight the impacts of advances in observations on the budget, the budget for overlapping periods (1993–2018) with AR6 and F2020 is shown in table S3 and fig. S2. The residual in this study is −0.05 mm year^−1^, which is much smaller than 0.40 mm year^−1^ in AR6 and 0.19 mm year^−1^ in F2020. It should be noted that the AR6 did not consider an ocean bottom deformation correction (*13*, *52*) or atmospheric water vapor. F2020 did consider ocean bottom deformation but not atmospheric water vapor. If both were considered, then the residual in AR6 and F2020 would be 0.58 and 0.26 mm year^−1^, respectively.

**Fig. 3.**
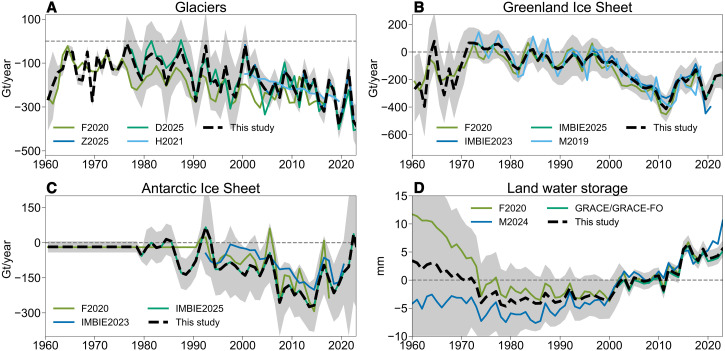
Comparison of barystatic components from different observation products. The black lines indicate data from this study, and shading represents a 90% confidence interval. Glacier comparisons (**A**) use F2020, Z2025 (*6*), D2025 (*83*), and H2021 (*90*). For the Greenland Ice Sheet (**B**), the comparison datasets are F2020, IMBIE2025, IMBIE2023, and M2019 (*91*). For the Antarctic Ice Sheet (**C**), we compare against F2020, IMBIE2025, and IMBIE2023. Land water storage (**D**) is evaluated relative to F2020 and M2024 (*70*).

Compared with AR6, the improved budget closure is primarily attributed to the updated Ice Sheet Mass Balance Inter-comparison Exercise (IMBIE) estimate (*7*) for the Antarctic Ice Sheet, along with revised peripheral glaciers contributions (Fig. 3C). The thermosteric sea level trend in this study is higher than that in F2020 by 0.19 mm year^−1^, owing to improved quality control (QC) used in IAPv4.2 (*53*). IAPv4.2 used a time-varying local climatology range accounting for local range change driven by long-term ocean changes, including more extreme values compared with a static threshold. It should be noted that the thermosteric sea level in AR6 did not incorporate recent advances in ocean temperature, such as instrument bias correction (*20*–*22*) and improved QC. In particular, the upper 700-m thermosteric sea level data in AR6 were derived using the altimetry sea level spatial pattern (*54*); it is unclear whether the gravitational and rotational fingerprints of the barystatic sea level change signal (*55*, *56*) could leak into the resultant estimate.

### Argo era (2005–2023)

**Fig. 4.**
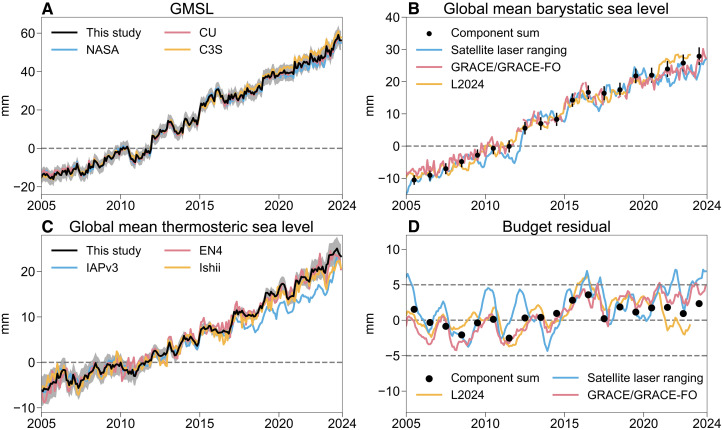
The time series of GMSL and individual contributors after 2005. The time series of GMSL as observed by satellite altimetry (**A**); global mean barystatic sea level based on GRACE/GRACE-FO, satellite laser ranging, L2024, and the component sum in this study (**B**); global mean thermosteric sea level from different gridded products (**C**); and the residual between GMSL and sum of contributions (**D**). The time series are corrected for the mean seasonal cycle relative to 2005–2015. The shadings represent a 90% confidence interval in this study.

Between 2005 and 2015, the monthly residuals of the sea level budget remain within ±5 mm, but residuals begin to increase after 2015, which indicates potential observational issues (*18*), as noted in the introduction. GMSL from various datasets exhibits differences (Fig. 4A), likely because the Jason-3 radiometer is subject to drift (*19*). This study adopts the WTC derived from climate records (*19*), which avoids the radiometer drift. The thermosteric sea level from IAPv4.2 is used, which can capture the ocean warming acceleration below 2000 m (*57*, *58*) through ensemble optimal interpolation (*24*). The Argo salinity observations after 2015 likely drift (*12*); however, this study focuses on thermosteric changes, given the limited contribution of salinity to global mean changes (*59*).

For barystatic sea level, estimates from the ensemble mean of GRACE/GRACE-FO (*60*–*62*), satellite laser ranging (*63*), and the sum of barystatic contributions in this study and in (*64*) (hereafter L2024) are used in this study (Fig. 4B). Regardless of which dataset, except for satellite laser ranging with high instrument noise, is combined with the IAPv4.2 thermosteric sea level, the budget residual after 2015 is mostly smaller than ±5 mm (Fig. 4D). Hence, by applying all necessary corrections for satellite altimetry and ocean temperature observations, the interannual GMSL budget can be closed within ±5 mm.

With the new datasets used in this study, during 2005–2023, altimetry data indicate a linear trend in GMSL of 3.94 ± 0.31 mm year^−1^, with thermosteric sea level contributing 1.55 ± 0.28 mm year^−1^. The increase in barystatic sea level is estimated at 2.24 ± 0.21 mm year^−1^ based on the component sum (Table 1). Estimates from GRACE/GRACE-FO and satellite laser ranging have similar results, showing a trend of barystatic sea level rise at 1.99 ± 0.16 mm year^−1^ and 2.13 mm year^−1^, respectively. Although the budget residual is almost smaller than ±5 mm, the trend residual is larger than 0.15 mm year^−1^. AR6 yields a smaller trend budget residual of 0.08 mm year^−1^; it does not mean the current observation system can close the budget fully because the ocean bottom deformation (*13*) and atmospheric water vapor are not included in the AR6 estimate, and the budget residual rose to 0.30 mm year^−1^ if they are accounted for.

Acceleration during 2005–2023 is strongly influenced by interannual variability, so our results should be interpreted with caution. The acceleration inferred from the altimetry is 0.84 ± 0.66 mm year^−1^ decade^−1^. If the interannual variability is removed through a multiple linear regression approach following Moreira *et al.* (*45*), then the acceleration is reduced to 0.75 mm year^−1^ decade^−1^. Thermosteric sea level is the major contributor with 1.15 ± 0.33 mm year^−1^ decade^−1^. This result is supported by observations of EEI. Observations from the Clouds and the Earth’s Radiant Energy System (*65*) show the trend of net radiative fluxes at the top of the atmosphere over 2005–2023 is 0.57 W m^−2^ decade^−1^, which is possibly driven by the reduction of anthropogenic aerosol emissions (*50*). If 90% energy is absorbed by the ocean, then the thermosteric sea level rise acceleration is 1.20 mm year^−1^ decade^−1^.

The barystatic sea level rate change is −0.35 ± 0.44 mm year^−1^ decade^−1^ based on the sum of components. This deceleration is mainly induced by the Greenland and Antarctic ice sheets changes (Table 1), at least partly driven by interannual variability associated with changing atmospheric teleconnection patterns (*33*). The barystatic sea level rate inferred from GRACE/GRACE-FO also shows a deceleration of −0.39 ± 0.27 mm year^−1^ decade^−1^. Both estimates satisfy the acceleration budget within uncertainty. Satellite laser ranging also confirms a deceleration of barystatic sea level.

## DISCUSSION

In this study, we account for updated community estimates and recent advancements in sea level observations and related components to reexamine budgets of the GMSL rise trend and acceleration across 1960 to 2023, 1993 to 2023, and 2005 to 2023. Although previous studies closed the GMSL budget (*16*, *36*, *66*), their results diverge (table S3) owing to different dataset choices. The up-to-date community estimates (*6*, *7*) reconcile differences among multiple estimate methods, mitigate the random errors induced by a single source, and reduce the differences from the dataset choice (see discussion in the “Data” and “Methods” sections).

For the linear trend analysis, the residual between observed GMSL and the sum of contributions has been reduced relative to AR6 and F2020, largely due to enhanced contributions from the community estimate of ice sheets, improved estimates of thermosteric sea level change accounting for instrument bias corrections, and the consideration of vertical land motion corrections beyond GIA models across different tide gauge reconstruction approaches. For example, the residual over 1993–2018 is 0.40 mm year^−1^ in AR6, whereas, in this study, it has been reduced to −0.05 mm year^−1^ (table S3). The trend budget residuals across the 1960–2023,1993–2023, and 2005–2023 periods are 0.17, −0.01, and 0.15 mm year^−1^; the large residual in the 1960–2023 period is induced by limited observations of land water storage in the 20th century. Incorporating the observational advances, the principal drivers for GMSL trend after 1960 are 43% thermosteric ocean expansion, 27% from glaciers, 15% from Greenland, 12% from Antarctic, and 3% from land water storage.

The acceleration budget in three eras can be closed within the respective uncertainties. The observed GMSL acceleration across the three eras remains relatively stable; however, the key drivers differ. The acceleration depends on the selected periods (Table 1) and is strongly influenced by interannual variability and how constant the drivers of each component acceleration are (such as the influence of volcanic eruptions on thermosteric sea level in the 1990s). For 1960–2023 and 1993–2023, the primary drivers are thermosteric sea level (41%) and Greenland Ice Sheet mass loss (35%), respectively. For 2005–2023, the strong acceleration of thermosteric sea level (1.15 mm year^−1^ decade^−1^) is partly compensated by the deceleration obtained for the barystatic component (−0.35 mm year^−1^ decade^−1^).

The acceleration budget closure achieved in this study complements previous studies, which considered ice sheets as the major driver (*32*), and implies that component accelerations are not stationary over time. In addition, the recent two decades’ budget indicates that increased EEI due to aerosol reduction and ice melting (*50*, *67*) can greatly influence GMSL. Some studies (*27*, *68*) use acceleration and trend derived from satellite altimetry to extrapolate future GMSL changes, and the results are close to the model-based projections, consistent with this study indicating that GMSL acceleration has been stable in time, at least since the 1960s (*26*). However, the relative contributions from individual component changes in response to different forcings, such as volcanic eruptions (*49*), EEI increase (*67*), and decadal variability (*33*).

Beyond trend and acceleration in three different periods based on annual data, this study also reassesses the monthly data of GMSL and the combination of barystatic and thermosteric sea level data after 2005 (Fig. 4). The residual of the monthly GMSL budget is reduced to ±5 mm.

Although this study reduces the residual of the budgets, there are several caveats. First, land water storage is a major uncertainty of the sea level budget since 1960 (Table 1). The estimate of groundwater is one of the most uncertain parts, because the global observation was not available until the launch of GRACE/GRACE-FO (*69*). Before that, the estimates are mainly derived from global hydrological models (*70*, *71*), but hydrological models cannot simulate human activities in heavily managed regions accurately (*72*). Its contribution to GMSL should still be considered in the future. Second, this study focuses on the global mean budget; however, whether the residual of the budget on basin scales can be reduced is unclear. The largest trend budget residuals in the Argo era occur in the North Atlantic and Indian Oceans (*17*, *73*). The regional budget will be explored in the future.

The reduction of the residual in the GMSL budget is achieved through advancements in observational techniques, indicating that the major contributors to the GMSL rise trend and acceleration have been successfully identified. The previous residual in the GMSL budget over 1971–2018, which is even larger than those from both the Greenland and Antarctic ice sheets, has been addressed by improved estimates of GMSL due to the consideration of vertical land motion estimates that go beyond GIA correction (*43*). Ultimately, maintaining and improving the Earth’s climate observation system is essential to resolving inconsistencies in GMSL acceleration.

## MATERIALS AND METHODS

### Data

#### Global sea level observations and reconstructions

This study uses two types of observations: tide gauge reconstruction and satellite altimetry. Similar to AR6, GMSL changes before 1993 are derived from tide gauge reconstructions (*36*, *39*, *40*), while those after 1993 are obtained from satellite altimetry observations. The budget based solely on tide-gauge reconstruction is provided in fig. S1.

Tide gauges, the main source of sea level information before the satellite era, measure relative sea level changes at points along the coast, providing a sparse yet unrepresentative sampling of the global ocean. Thus, tide gauge observations are usually adjusted for a variety of factors that contribute to spatial variability in sea level, before estimating GMSL changes. The consideration of this spatial variability is handled differently in the various reconstructions and for different contributing factors, either through ad hoc corrections or through the inclusion of spatial covariance information. The main approaches include the Kalman smoother, RSOI, and the virtual station technique. This study integrates the three most recent reconstructions from these three categories (*36*, *39*, *40*) with a Monte Carlo approach (*36*), which covers the main reconstruction methods. All reconstructions take into account biases of local tide gauge records through spatial variability in vertical land motion, barystatic gravitational and rotational fingerprints, steric sea level, and inverse barometer effects (*41*, *42*).

Satellite altimetry has provided geocentric sea level change estimates since 1993, with precise, near-global measurements at 10-day frequency. Here, the altimetry-based GMSL is adopted from the mean of datasets from Copernicus Climate Change Service (C3S) (*74*), University of Colorado, and NASA (*75*). The estimate is corrected for TOPEX-A drift (*66*), GIA effect (*76*), ocean bottom deformation due to present day ice melting (*13*), and WTC based on climate records (*19*). Uncertainties considered include offsets among successive missions, trend uncertainties in the TOPEX-A and TOPEX-B instruments, altimetry noise WTC, and orbital errors. Further details on the uncertainty of altimetry can be found in (*47*).

#### Thermosteric sea level

The focus is on the thermosteric sea level and neglects the halosteric contributions. This is an acceptable approximation because regional compensation leads to weak global-scale changes (*59*), and the potential bias in salinity data persists (*12*). The thermosteric sea level used in this study is calculated using the IAPv4.2 temperature monthly (*10*) and salinity climatology dataset at a 1° by 1° resolution covering the upper 6000 m (119 levels). The raw ocean temperature observations come from Nansen bottles (predominantly used 1900–1940), Mechanical Bathythermographs (MBT; dominating 1940–1970), eXpendable BathyThermograph (XBT; dominating 1970–2001), Argo floats (widely adopted after 2005), marine mammal dataloggers (accounting for nearly half of profiles in high latitudes after 2005), and conductivity-temperature-depth profilers (*10*). Certain instruments are prone to systemic biases and are an important source of uncertainty in the sea level budget. In the IAPv4.2 ocean temperature product, the biases in Nansen bottles (*21*), MBT (*22*), XBT (*23*), and marine mammal dataloggers (*20*) have been adjusted.

Besides systemic biases, the incorrect observation records induced by equipment failures, external interference, and transcoding errors are another source of errors. The QC system has been used to identify spurious records through a set of checks, and an updated version is used in the IAPv4.2 product (*53*). The advances include: (i) accounting for non-Gaussian distributions in local climatological thresholds instead of traditional fixed-SD (Gaussian) methods, thereby better removing spurious data in light of realistic climatic signals associated with eddies, extreme events, fronts, and pronounced warming regions; (ii) consideration of the influence of water mass properties and ocean-land topography to define climatological thresholds, enhancing the accuracy and sensitivity of the QC process; (iii) incorporation of resolution-dependent vertical gradient threshold checks, enabling precise detection of anomalous vertical profile shapes; and (iv) inclusion of targeted QC measures tailored to specific-instrument-related errors, such as XBT.

For spatial interpolation (mapping) to create gridded temperature products, the IAP dataset uses remote observations to inform data-sparse regions guided by dynamical covariance from climate model simulations (*24*), ensuring reconstruction of interannual to multidecadal scale signals while overcoming systemic biases in existing techniques (especially “no observation, no signal” paradigm and isotropic reconstruction artifacts). The integration of ocean dynamical information derived from multimodel outputs, rather than reliance on geometric interpolation, substantially enhances reconstruction accuracy. Additionally, a resampling test developed to evaluate the approach demonstrates that the IAP dataset is capable of representing ocean heat content variability across multiple spatiotemporal scales (*77*).

The uncertainty in the thermosteric sea level is based on the mapping framework (*24*) and accounts for instrumental, sampling, and mapping errors, as well as QC. For comparison, the thermosteric sea level is also calculated from the Ishii ocean objective analysis product (*78*), EN4 ocean objective analysis product from the UK Met Office Hadley Centre (*79*), and IAPv3 (*80*) for the upper 2000 m salinity and temperature data. A linear trend of 0.1 mm year^−1^ is added to account for changes below 2000 m based on repeated hydrographic sections (*81*). The global mean thermosteric sea level changes are calculated within 66°S to 66°N.

#### Glacier mass loss

The glacier estimates before 2000 in this study rely on a combination of model reconstructions (*82*) and hybrid observations (*83*), whereas, after 2000, they are derived from the community estimates provided by GlaMBIE (*6*). Uncharted glaciers are included (*11*). The peripheral glaciers are not considered in the glaciers because they are included with the Antarctic and the Greenland ice sheets in this study.

Model reconstruction and in situ observations are the most widely used methods for evaluating glacier mass loss before 2000 (*16*, *36*). Model reconstructions can be used to estimate glacier mass change rates from atmospheric forcing variables (e.g., temperature and precipitation) and are complete in time and space, complementary to the spatially sparse observations. The hybrid observations (*83*) are used in this study, complementing in situ observations (*5*), integrating glacier elevation changes to increase spatial coverage. In addition, the model reconstructions (*82*) in this study refine those used in AR6 and F2020 with boundary and initial conditions from the updated observations. The uncertainty is generated with the Monte Carlo method, which accounts for the interior uncertainty from individual products and structural uncertainty between two datasets.

Unlike the exclusive use of digital elevation model (DEM) in AR6 and GRACE/GRACE-FO in F2020, this study adopts the community datasets, which include results from glaciological observations, DEM, altimetry, and GRACE/GRACE-FO (*6*). The uncertainties are sourced from interior input datasets, heterogeneity, and structural differences among methods and glaciers.

#### Ice sheets mass loss

This study adopts the IMBIE estimates (*7*) for the Antarctic Ice Sheet (1979–2023) and the Greenland Ice Sheet (1972–2023). Antarctic Ice Sheet changes before 1979 are derived from Frederikse *et al.* (*36*), while Greenland Ice Sheet changes before 1972 are estimated using the input-output method (*30*). All datasets except for Mankoff *et al.* (*30*) have considered peripheral glaciers; thus, the Greenland peripheral glacier estimate from Dussaillant *et al.* (*83*) is added.

Estimates of the Antarctic Ice Sheet before 1979 are limited; here, we adopt a compilation from Frederikse *et al.* (*36*), which assumes a contribution of 0.05 ± 0.04 mm year^−1^. Greenland Ice Sheet changes before 1972 are obtained from the input-output method (*30*), which first includes basal mass balance.

IMBIE estimates are sourced from 50 independent datasets (27 for Greenland Ice Sheet and 23 for Antarctic Ice Sheet) based on the input-output method, satellite altimetry, or GRACE/GRACE-FO. Input datasets are corrected for biases such as firn compaction and GIA, converted to consistent units, and combined to produce annual mass-change time series with quantified uncertainties. The uncertainty includes interior uncertainty from a single method (such as density assumptions, GIA, and model uncertainty) and structural uncertainty.

#### Land water storage

Land water storage in this study before 2003 is based on a global hydrological model (*70*) and a composite dataset from F2020, following AR6. After 2003, the estimates are taken from GRACE/GRACE-FO (*60*). It should be noted that glaciers larger than 100 km^2^ are excluded to avoid double counting.

The composite dataset from F2020 combines natural and anthropogenic changes from different sources. The natural changes are estimated on the basis of a statistical reconstruction model trained with GRACE observation results (*84*). The anthropogenic changes are considered on the basis of the reservoir impoundment (*85*) and groundwater depletion (*71*, *86*). Water – Global Assessment and Prognosis (*70*) is also used to complement the estimate F2020, with complete spatial coverage.

#### Atmospheric water vapor

The atmospheric water vapor estimates in this study after 1993 are taken from the European Centre for Medium-Range Weather Forecasts fifth reanalysis (ERA5) (*87*). The changes before 1993 are not considered due to inhomogeneities and limited observation for the analyses (*38*). ERA5 assimilates multisource data, including ground-based measurements and satellite observations, using a 4D-var ensemble data assimilation system with a global spatial resolution of 0.5° by 0.5°. ERA5 agrees well with special sensor microwave imager (SSM/I) observations (*38*), and the penalized maximal F test (*37*) shows no jump point after 1993. The estimates are based on a 10-member ensemble, described by its mean and 1.65 times its SD (i.e., 90% confidence intervals).

#### Ocean mass from GRACE/GRACE-FO

GRACE/GRACE-FO provide direct observations of ocean mass from monthly gravity field solutions since 2002. To obtain the ocean mass change from mascon data, the spatial mean of the GAD product over ocean is removed. Three groups of GRACE/GRACE-FO products from different processing centers are used in this study: Jet Propulsion Laboratory RL06.1Mv3 (*60*), the Center for Space Research RL06.2 (CSR) (*62*), and the Goddard Space Flight Center RL06v2.0 (*61*). The global mean ocean mass is calculated between 66°S and 66°N.

### Methods

The trend and acceleration (calculated as twice the quadratic coefficient) in this study are derived using OLS regression. The uncertainty estimates follow the methodology outlined by Ablain *et al.* (*25*). A brief introduction to the approach is provided below [see (*25*) for more details].

The linear regression model for the estimation of trends and accelerations

y=Xβ+ϵ (1)

where y is an *n* × 1 vector representing the time series data. X is an *n* × 3 matrix, where *n* is the length of the time series. The first column of X contains only ones (representing the constant term), the second column contains the time vector (representing the linear term enabling the derivation of the trend information from the time series), and the third column contains the square of the time vector (representing the squared term enabling the derivation of the acceleration information from the time series). β is a 3 × 1 vector of unknown parameters to be estimated, specifically the intercept, the linear term (which contains the trend information), and the quadratic term (which contains the acceleration information). ϵ is an *n* × 1 vector of disturbances and errors. OLS is used to estimate the parameters β, providing an unbiased best-fit of the parameters through the time series data by minimizing the sum of squared residuals.

The uncertainty of the estimates follows Ablain *et al.* (*25*), where $\hat{\beta }=\;N\;\left[\;\beta ,\;{\left(\;X^{t}X\;\right)}^{-1}\;\left(X^{t}\;\sum \;X\right)\;{\left(\;X^{t}X\;\right)}^{-1}\;\right]$. ∑ is the error variance-covariance matrix. The error variance-covariance matrices of GMSL from the tide gauge reconstruction, thermosteric sea level, ocean mass inferred from GRACE/GRACE-FO, and atmospheric water vapor are derived with an ensemble approach where the ensemble time series is directly used to calculate the covariance between each time series [as in (*88*)]. The error variance-covariance matrices of GMSL based on satellite altimetry, glaciers, Greenland Ice Sheet, Antarctic Ice Sheet, and land water storage are constructed through a decomposition of each uncertainty source [section 4 of (*25*)].

The uncertainty of trend and acceleration is calculated as follows

$$\left[\begin{array}{ccc} {\left(\frac{\text{er}r_{\text{acceleration}}}{2}\right)}^{2} & \cdots & 0\\ \vdots & {\text{err}}_{\text{trend}}^{2} & \vdots \\ 0 & \cdots & {\text{err}}_{\text{constant}}^{2} \end{array}\right]={\left(X^{t}X\right)}^{-1}\left(X^{t}\sum X\right){\left(X^{t}X\right)}^{-1}$$ (2)

The terms err_trend_ and err_acceleration_ are the uncertainties of the trend and acceleration, respectively. In this study, we provide uncertainties with a 90% confidence interval (i.e., 1.65 err_trend_ and 1.65 err_acceleration_ assuming a normal distribution).

Although the OLS method is widely used in climate research, the results can be substantially affected by interannual variability (*45*, *89*), and, hence, we included the results with the interannual variability of GMSL removed by regressing it against the multiple climate indices following Moreira *et al.* (*45*). The results are summarized in table S1.

To complement the OLS, alternative methods were used to estimate the GMSL trend. For instance, the AR6 adopts a simple method (called “Delta method” in this study) (*35*), where trends are calculated as the difference between the start and end points of the period divided by the time length. Another method is the LOWESS method (*28*). By applying LOWESS to the time series with a 25-year window (equivalent to an effective 15-year smoothing), the trend is calculated as the difference between the first and last years of the smoothed series. This approach effectively reduces the influence of interannual variability and start/end point biases while offering a flexible framework for estimating localized trends. The GMSL trend estimate results based on different methods are summarized in table S1.
