# Supplementary material for: Improved closure of the global mean sea level budget from observational advances since 1960
Source: Sci Adv. 2026 May 20;12(21):eaea0652. doi: 10.1126/sciadv.aea0652 (PMC13189096; doi:10.1126/sciadv.aea0652)
Supplement: Supplementary file 1 — Figs. S1 to S3 Tables S1 to S3 [file sciadv.aea0652_sm.pdf]

Science Advances
AAAS

Supplementary Materials for

Improved closure of the global mean sea level budget from observational advances since 1960

Huayi Zheng *et al.*

Corresponding author: Lijing Cheng, chenglij@mail.iap.ac.cn

Sci. Adv. **12**, eaea0652 (2026)
DOI: 10.1126/sciadv.aea0652

This PDF file includes:

Figs. S1 to S3
Tables S1 to S3

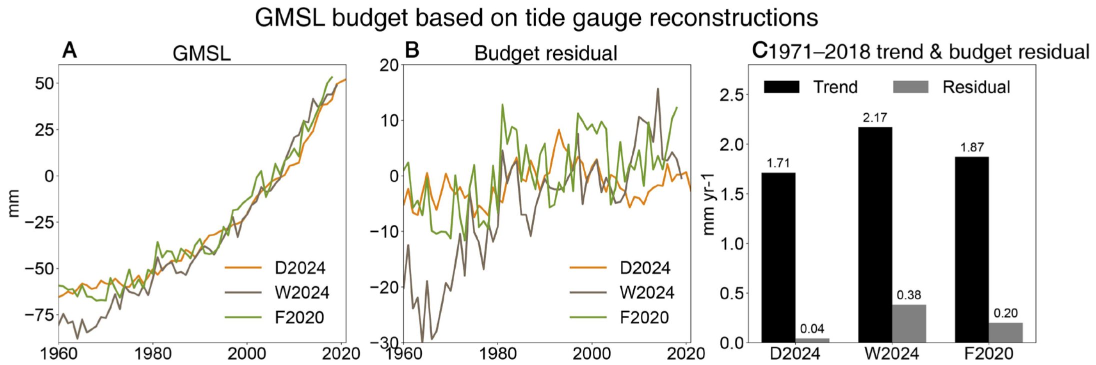

Fig. S1. GMSL budget based on tide gauge reconstructions. (**A**) The time series of GMSL derived from different reconstructions (36, 39, 40). (**B**) The time series of budget residuals with different reconstructions. (**C**) The 1971-2018 trend and budget residual for different reconstructions.

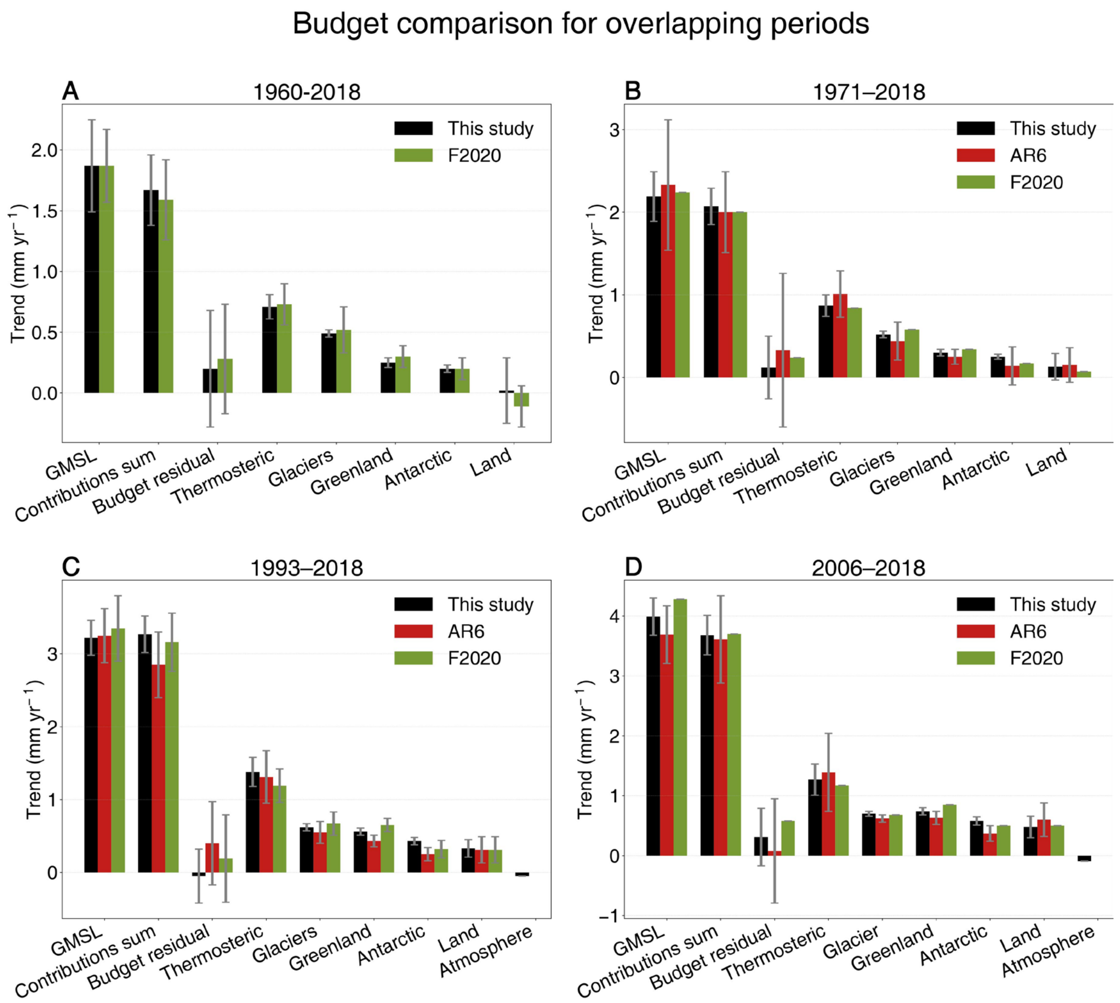

Fig. S2. Comparison of the GMSL budget with AR6 and F2020 estimates over overlapping periods (1960–2018, 1971–2018, 1993–2018, and 2006–2018). The error bar represents 90% confidence interval.

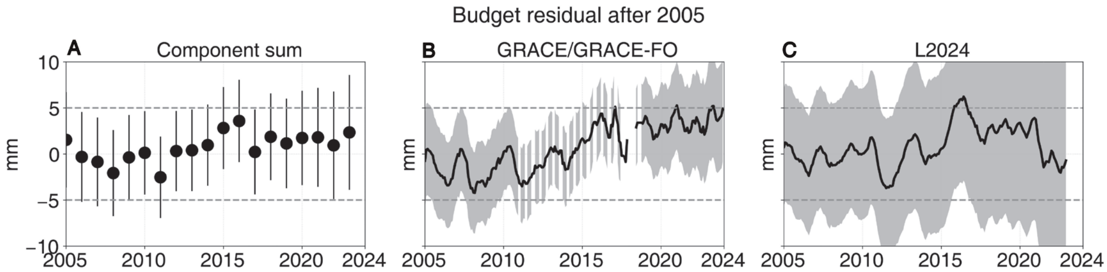

Fig. S3. The budget residual after 2005. The GMSL is the ensemble mean of CU, AVISO, and NASA with ocean bottom deformation correction and WTC based on the climate record. The thermosteric sea-level is derived from IAPv4.2. The budget residuals with respect to barystatic sea-level are shown based on the component sum (left), GRACE/GRACE-FO (middle), and L2024 (right). The shaded areas and error bars denote the 90% confidence intervals.

Table S1. The datasets used in this study with key improvements.

	Dataset	Key improvements
Altimetry-based sea-level	NASA, C3S, and CU with ocean bottom deformation correction (*13*) and wet troposphere correction based on climate data records (*19*)	1) Spurious drifts in the on-board radiometer are corrected.<br>2) Ocean bottom deformation is included.
Tide gauge reconstruction	Dangendorf et al. (*40*), based on Kalman smoother combined with reduced spatial optimal interpolation without EOF0<br>Frederikse et al. (*36*), based on virtual-station method<br>Wang et al. (*39*), based on reduced spatial optimal interpolation with EOF0	Local observations in combination with prior information on contributing processes are used to account for local variability.
Thermosteric sea-level	IAPv4.2 (*10*)	1) Newly identified biases in bottle and APB data are corrected; XBT and MBT bias corrections are updated.<br>2) The advanced quality-control system is used. One advance is using time-varying climatological range to identify outliers accounting for the impact of climate change in data distributions.<br>3) The mapping method utilizes dynamical covariance from climate

		model simulations to inform reconstruction in data-sparse regions. The performance is thoroughly evaluated with a resampling method.
Glaciers	1960–2000: observation derived from Dussaillant et al. (*83*) and ensemble-based model reconstruction from Malles and Marzeion (*82*) are combined with Monte Carlo approach<br><br>2000–2023: Zemp et al. (*6*), covering 19 regions based on 233 records from 35 research teams, including results from glaciological measurements, digital elevation model differencing, altimetry, and gravimetry<br><br>1960–2023: Uncharted glaciers are included (*11*).	1) Observation datasets adopt geostatistical modeling for interpolation to increase the satellite temporal resolution from decadal to yearly.<br><br>2) Model reconstruction is driven by 11 different forcing datasets. And the boundary and initial conditions are sourced from updated observations.<br><br>3) The 2000–2023 estimates are derived from the combination of multiple methods rather than DEM only in AR6.
Greenland Ice Sheet	1960–1973: Mankoff et al. (*30*), based on the input–output method<br><br>1973–2023: Otosaka et al. (*7*), synthesised from 27 datasets using satellite altimetry, satellite gravimetry, or the input–output method	1) The estimate before 1973 is based on an input-output model, which includes basal mass balance.<br><br>2) The community estimate from IMBIE is extended to 1973-2023.
Antarctic Ice Sheet	1960–1979: Frederikse et al. (36), based on compilation	The community estimate from IMBIE is extended to 1979-2023.

	1979–2023: Otosaka et al. (*7*), synthesised from 27 datasets based on satellite altimetry, satellite gravimetry, or the input–output method	
Land water storage	1960–2003: observation-based estimate (*36*) and hydrological model WaterGAP v2.2e (*70*) are merged through Monte Carlo approach<br><br>2003–2023: JPL GRACE/GRACE-FO	Land water storage from GRACE/GRACE-FO is extended to 2023.
Atmospheric water vapor	1993–2023: ERA5 (*87*)	

Table S2. Trends (mm yr^{-1}) for GMSL based on different methods.

	1960–2023	1993–2023	2005–2023
OLS	2.06	3.41	3.94
OLS (removing inter-annual variability)		3.48	3.95
Delta method	2.05	3.49	3.79
LOWESS method	2.06	3.21	3.79

Table S3. The budget comparison with AR6 and F2020. F2020 does not provide a budget for 1971–2018 and 2006–2018 directly; thus, we calculate the budget based on their time series. The uncertainty in F2020 is based on ensembles; thus, the uncertainty for 1971–2018 and 2006–2018 is not provided here due to data availability.

		1960–2018 trend (mm yr^{-1})	1971–2018 trend (mm yr^{-1})	1993–2018 trend (mm yr^{-1})	2006–2018 trend (mm yr^{-1})
Thermosteric	This study	0.71 ± 0.10	0.87 ± 0.13	1.38 ± 0.20	1.27 ± 0.26
	AR6		1.01 ± 0.28	1.31 ± 0.36	1.39 ± 0.65
	F2020	0.73 ± 0.17	0.84	1.19 ± 0.23	1.17
Glaciers	This study	0.49 ± 0.03	0.52 ± 0.04	0.62 ± 0.05	0.70 ± 0.04
	AR6		0.44 ± 0.23	0.55 ± 0.15	0.62 ± 0.06
	F2020	0.53 ± 0.19	0.58	0.67 ± 0.16	0.68
Greenland Ice Sheet	This study	0.25 ± 0.04	0.30 ± 0.04	0.56 ± 0.05	0.74 ± 0.06
	AR6		0.25 ± 0.09	0.43 ± 0.08	0.63 ± 0.11
	F2020	0.30 ± 0.09	0.34	0.65 ± 0.09	0.85
Antarctic Ice Sheet	This study	0.20 ± 0.03	0.25 ± 0.03	0.43 ± 0.05	0.58 ± 0.07
	AR6		0.14 ± 0.23	0.25 ± 0.09	0.37 ± 0.13
	F2020	0.14 ± 0.09	0.17	0.32 ± 0.12	0.50
Land water storage	This study	0.02 ± 0.27	0.13 ± 0.16	0.33 ± 0.12	0.48 ± 0.18
	AR6		0.15 ± 0.21	0.31 ± 0.18	0.60 ± 0.28
	F2020	-0.11 ± 0.17	0.07	0.31 ± 0.18	0.50
Atmospheric water vapor	This study			-0.05 ± 0.00	-0.09 ± 0.00
Sum of contributions	This study	1.67 ± 0.29	2.07 ± 0.22	3.27 ± 0.25	3.68 ± 0.33
	AR6		2.00 ± 0.49	2.85 ± 0.44	3.61 ± 0.73
	F2020	1.59 ± 0.33	2.00	3.16 ± 0.40	3.7
GMSL	This study	1.87 ± 0.38	2.19 ± 0.30	3.22 ± 0.24	3.99 ± 0.31
	AR6		2.33 ± 0.79	3.25 ± 0.37	3.69 ± 0.48
	F2020	1.87 ± 0.30	2.24	3.35 ± 0.45	4.28
Budget residual	This study	0.20	0.12	-0.05	0.31
	AR6		0.33	0.4	0.08
	F2020	0.28	0.24	0.19	0.58
